# Impact of local delivery of allogeneic chondrocytes on the biological response and healing of the sternum bones after sternotomy

**DOI:** 10.1038/s41598-023-43255-y

**Published:** 2023-09-25

**Authors:** Agata Krauze, Agnieszka Fus-Kujawa, Karolina Bajdak-Rusinek, Dorota Żyła-Uklejewicz, Carlos Fernandez, Ilona Bednarek, Sabina Gałka, Łukasz Sieroń, Edyta Bogunia, Mateusz Hermyt, Jerzy Nożyński, Krzysztof Milewski, Piotr Czekaj, Wojciech Wojakowski

**Affiliations:** 1 Center for Cardiovascular Research and Development, American Heart of Poland SA, 43-450 Ustroń 1, Poland; 2 Department of Medical Genetics, Faculty of Medical Sciences in Katowice, Medical University of Silesia, Medykow 18 Street, 40-752 Katowice, Poland; 3 Department of Biotechnology and Genetic Engineering, Faculty of Pharmaceutical Sciences in Sosnowiec, Medical University of Silesia, 40-055 Katowice, Poland; 4 Department of Cytophysiology, Chair of Histology and Embryology, Faculty of Medical Sciences in Katowice, Medical University of Silesia, Medykow 18 Street, 40-752 Katowice, Poland; 5 Department of Histopathology, Silesian Centre for Heart Diseases, 41-800 Zabrze, Poland; 6 Division of Cardiology and Structural Heart Diseases, Medical University of Silesia, Katowice, Poland

**Keywords:** Cardiology, Medical research

## Abstract

Median sternotomy is the surgical method of choice for many procedures where one of the main problems is the long post-operative wound healing process leading to sternal dehiscence and the development of infection. This leads to prolonged hospital stay and increased mortality due to post-operative complications. A promising solution seems to be the use of allogeneic chondrocytes for wound treatment, whose properties in the field of cartilage reconstruction are widely used in medicine, mainly in orthopedics. In the present study, we investigated the effect of local delivery of allogeneic chondrocytes on the biological response and healing of the sternum after sternotomy. We optimized the culture conditions for the isolated chondrocytes, which were then applied to the sternal incision wound. Chondrocytes in the culture were assessed on the basis of the presence of chondrocyte-specific genes: Sox9, Aggrecan and Collagen II. In turn, the histopathological and immunohistochemical evaluation was used to assess the safety of implantation. In our work, we demonstrated the possibility of obtaining a viable culture of chondrocytes, which were successfully introduced into the sternal wound after sternotomy. Importantly, implantation of allogeneic chondrocytes showed no significant side effects. The obtained results open new possibilities for research on the use of allogeneic chondrocytes in the process of accelerating wound healing after median sternotomy.

## Introduction

The development of cardiac surgery has made median sternotomy the most commonly used method of access to the mediastinal organs^1^. This approach is used by the majority of cardiac surgeons, as well as thoracic surgeons in the case of interventions performed due to anterior mediastinal tumor and bilateral operations, such as bullae resection, reduction of lung volume in the treatment of generalized emphysema, or simultaneous resection of metastases in both lungs^2–5^. Median sternotomy procedures are burdened with serious limitations, such as problems with the healing of the sternum and accompanying complications^6–8^. This includes sternal dehiscence, occurring in 0.2–5% of cases^9–11^.

An unstable sternum is a serious and potentially dangerous complication that can lead to respiratory failure, chest pain, mediastinal infection, and prolonged hospitalizations^10–14^.

The causes of delayed sternum healing are: lack of primary synostosis, poor wound healing or premature mobilization. In turn, mortality due to sternal instability used to reach 50%, but medical progress and the development of new technologies have contributed to a significant reduction to about 10%, which is still a serious problem^15,16^. In addition, the development of these complications is influenced by many risk factors associated with the patient's comorbidities, such as obesity, osteoporosis, heart failure, as well as his lifestyle and the surgery performed^4,14,17^.

Various methods are used to accelerate the healing of sternum wounds, reducing the risk of post-operative complications. These methods are constantly being improved^15,18,19^. Among them, there are innovative techniques involving the use of specially prepared ribs and a scapula to function like a previously removed sternum^20,21^. Despite the promising results of these methods, they are still very invasive and carry a high risk of complications and mortality. Sternum dehiscence remains a significant problem and there are no effective methods to prevent it^15,19^. In the face of these issues, it seems clear that the problem affects a wide and diverse population.

The solution to this problem could be the use of allogeneic chondrocyte cultures taken from the sternum cartilage. The chondrocyte culture technique is widely used in orthopedics for the treatment of knee defects, where autologous chondrocytes are obtained directly from the injured patient's knee joint^22–25^. This technique produces impressive results. After six months, the lesion is not visible in endoscopic control, it is completely overgrown and covered with cartilage^23^.

Herein, we have proved that it is possible to obtain a viable culture of chondrocytes, that have been successfully introduced into the sternal wound after sternotomy without causing side effects.

## Results

### Swine chondrocytes demonstrate expression of specific markers

**Figure 1:**
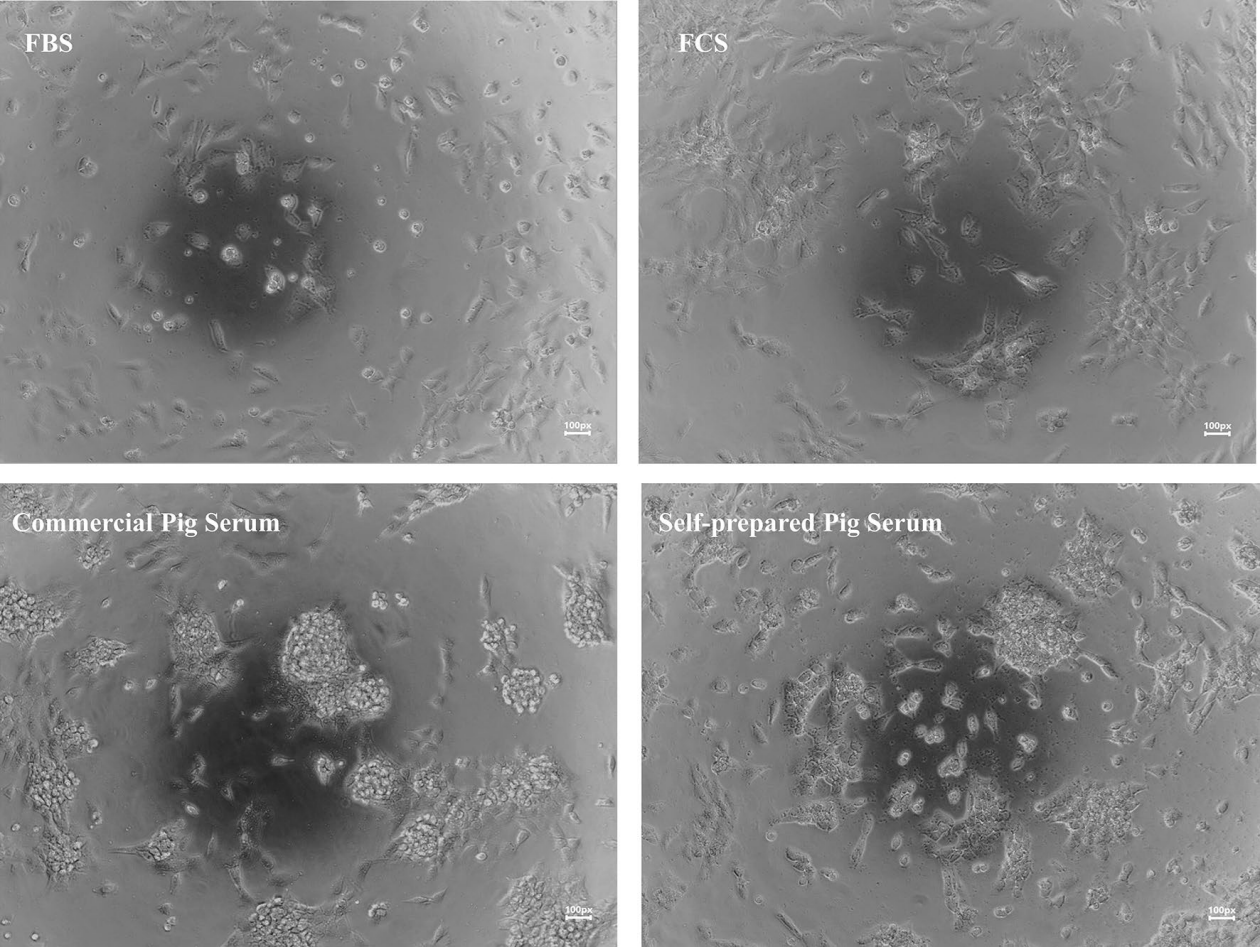
Swine chondrocytes morphology. Cells were cultured in four different media serum: Fetal bovine serum (FBS), Fetal calf serum (FCS), self-prepared pig serum and commercial pig serum. Cell morphology was analyzed at the same time points. Images were taken under an inverted light microscope (Delta Optical, Belgium). Scale bars = 100 µm.

Swine chondrocytes were successfully isolated from domestic pigs (*Sus scrofa domesticus*) xiphoid cartilage. The cells were cultured in 4 different media serum in order to select the one in which the cell proliferation was the highest. After the adhesion time, swine chondrocytes acquired a specific phenotype (Fig. 1).

**Figure 2:**
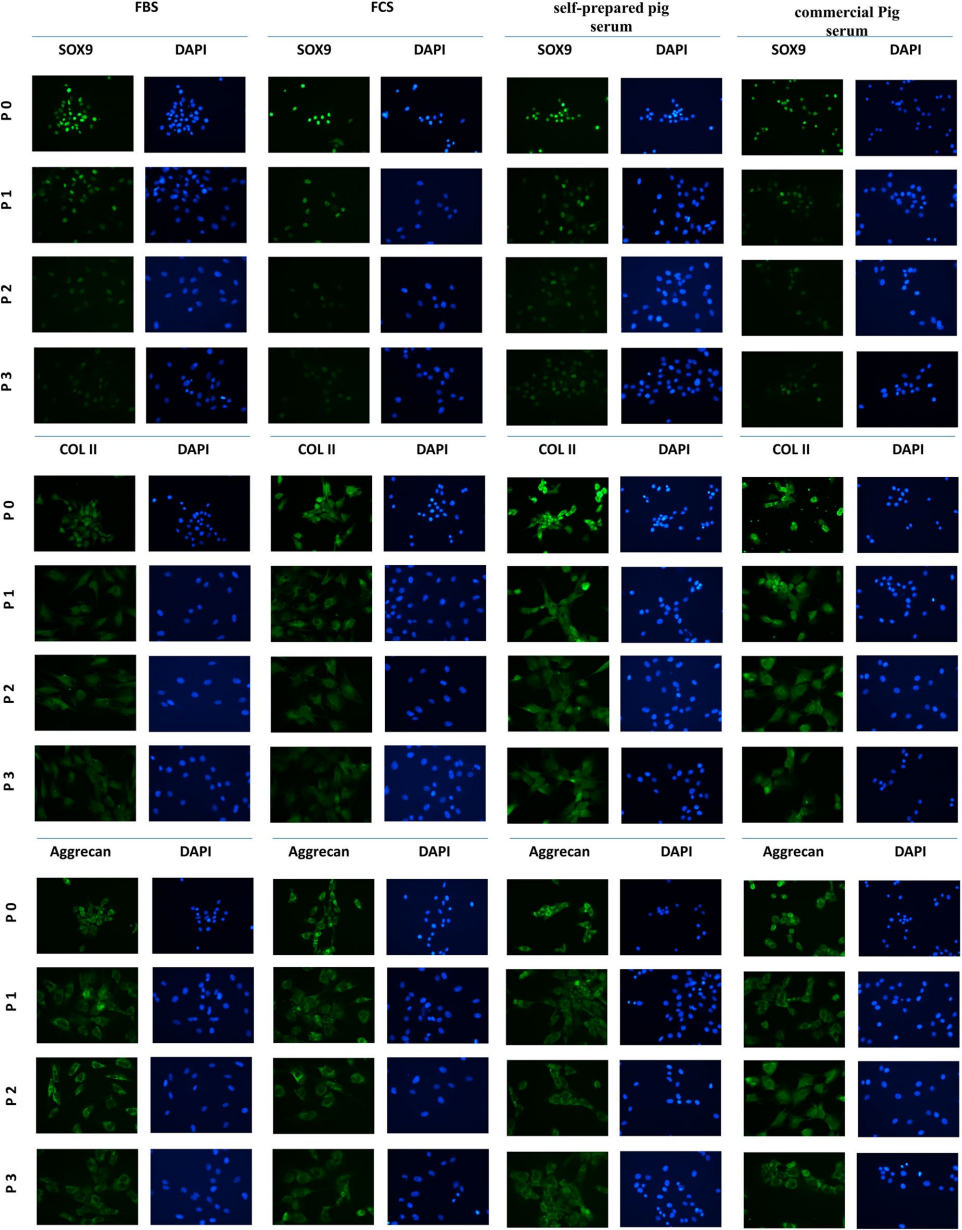
Immunocytochemical analysis of Sox9, Collagen II and Aggrecan expression in swine chondrocytes. Representative pictures show the expression of surface markers in all tested serum (FBS, FCS, commercial Pig serum and self-prepared pig serum) in four passages (0, 1, 2, 3). Nuclei were stained with DAPI. Pictures were taken under a fluorescent microscope (Nikon ECLIPSE Ti, USA).

We then confirmed the phenotype of the obtained porcine chondrocytes by immunocytochemical (ICC) analysis. Our results revealed that the established cells were positive for specific chondrogenic markers: Sox9, Collagen II and Aggrecan (Fig. 2).

**Figure 3:**
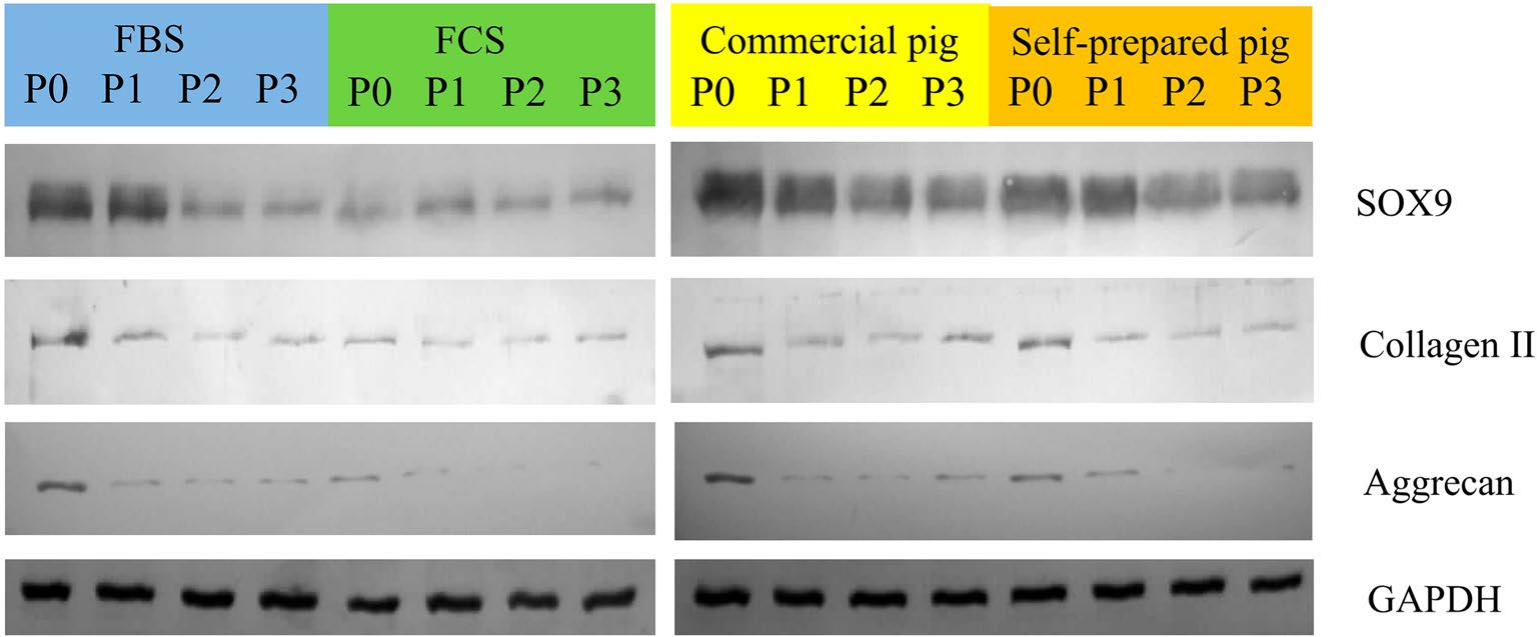
Identification of Sox9, Collagen II and Aggrecan in swine chondrocytes cultured in different serum at passage 0, 1, 2 and 3. In all tested serum media we identified proteins of interest. The blots were cut before hybridization with antibodies. GAPDH was used as a loading control. Original blots are shown in the Supplementary Materials; Fig. S1.

In order to further assess the quality of swine chondrocytes, we checked the presence of Sox9, Collagen II and Aggrecan in the analysed cells. We have proven that chondrocytes cultured in all tested media serum reveal the presence of all identified proteins. The highest amount of protein was observed in cells cultured in commercial pig serum and self-prepared pig serum (Fig. 3). Evaluation of the functionality of porcine chondrocytes is crucial for their use in in vivo therapies and regenerative medicine.

**Figure 4:**
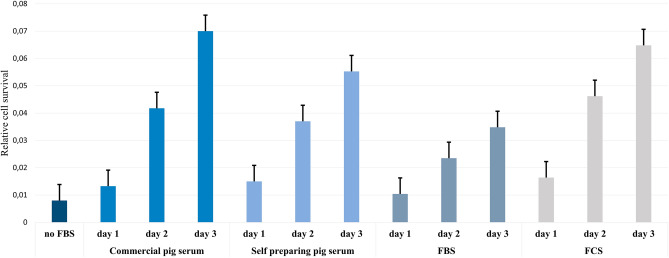
Cell proliferation assay. The X-axis indicates the variant of the serum used on the following days (1, 2 and 3). The Y-axis indicates relative cell survival. Values are mean ± SD (n = 3).

To use chondrocytes with the highest proliferation rate, we evaluated the proliferative capacity of swine chondrocytes cultured in 4 different serum media. For this purpose, we performed the MTT assay at 4 time points (Fig. 4). The results showed that chondrocytes cultured in commercial and self-prepared pig serum had the highest survival ratio. Considering this, we selected this serum for further analysis and in vivo studies.

### Swine chondrocytes display expression of specific chondrogenic genes

**Figure 5:**
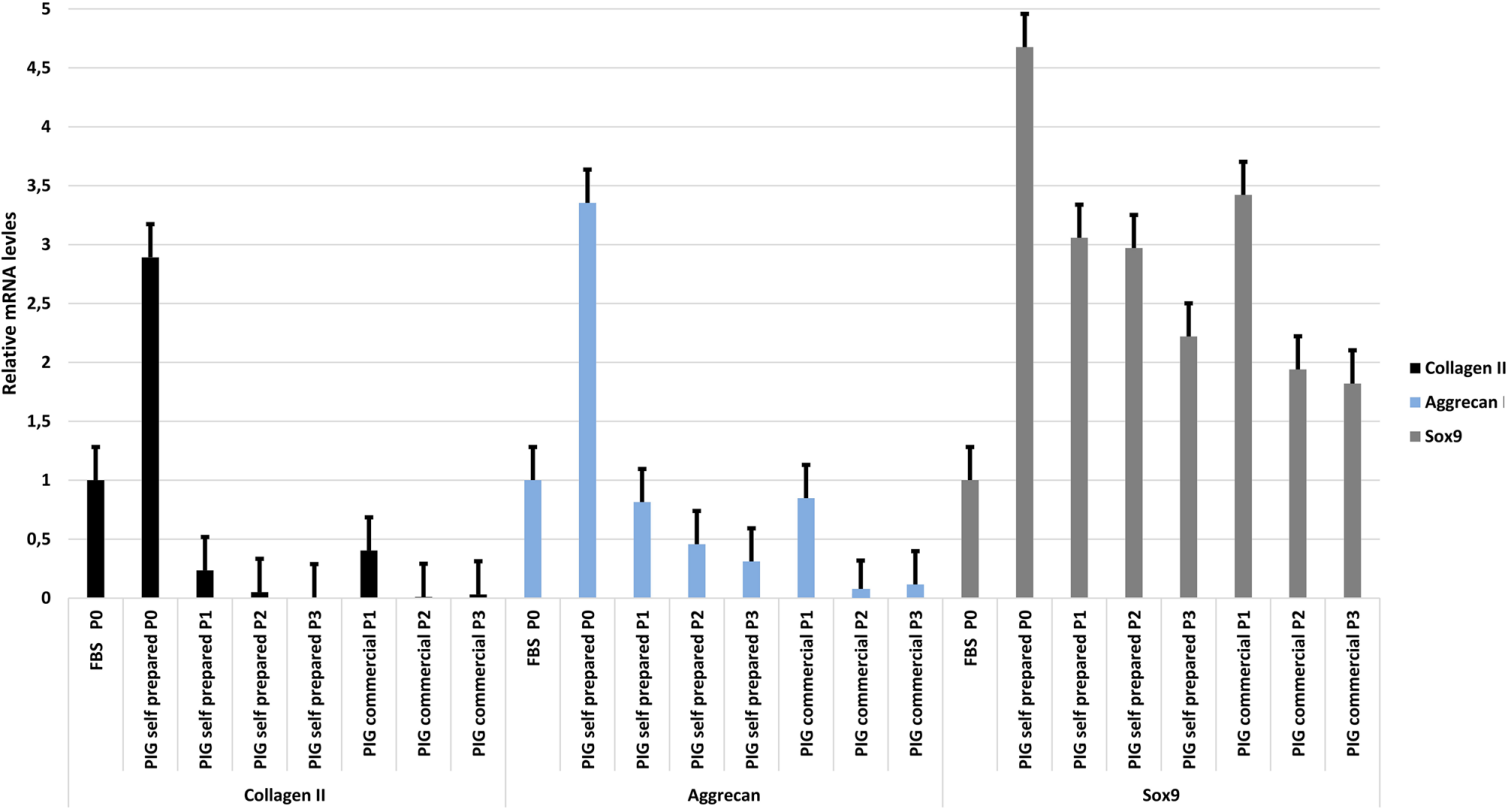
RT‐qPCR expression analysis of Sox9, Collagen II and Aggrecan in swine chondrocytes. Gene expression was evaluated at different time points: at passage 0, 1, 2 and 3. GAPDH was used as loading control. Values are mean ± SEM (n = 3).

To confirm the functionality of the obtained cells, we performed an analysis of chondrocyte-specific gene expression. Our previous analyzes showed that the highest level of proliferation was obtained when chondrocytes were cultured in the commercial and self-prepared pig serum. Therefore, the relative gene expression of chondrocytes cultured in two different serum media and at different passages was compared (Fig. 5).

We observed the highest gene expression for commercial and self-prepared pig serum at passage 0 in all analysed genes. Sox9 expression was higher in all tested passages (0–3) compared to Aggrecan and Collagen II, where we observe a decrease in gene expression already at passage 1.

### Morphological and biochemical analysis of blood confirms the safety of allogeneic chondrocyte implantation

To assess the safety of allogeneic chondrocyte implantation, we analyse blood samples taken before the procedure, 3 and 6 weeks after the procedure in both, the control and study groups. Administration of a new medical glue to enhance the healing process of bone structures did not induce any measurable septic reaction in serum samples in the study group. Results from TNF, IL-1 and IL-6 taken at baseline, and at 3 and 6 weeks of follow up showed no statistically significant variation and were comparable between time points and groups (Table 1).

**Table 1:** Blood analysis in the study group after 3 and 6 weeks of observation.

Group		TNF (pg/ml)	IL-1α (pg/ml)	IL-6 (pg/ml)
3 Weeks	Mean	223.96	1038.40	38.35
	SD	133.71	564.04	60.85
	p =	0.70	0.17	0.67
6 Weeks	Mean	242.58	1360.57	236.13
	SD	167.70	812.63	352.17
	p =	0.59	0.85	0.33

CRP levels were statistically significant after 3 weeks of observation (p = 0.01) with lower values in the control group compared to the study group. After 6 weeks of observation, this difference was no longer noticeable (p > 0.05). CBC and biochemistry were normal in both groups with some minor deviations from reference ranges with no clinical relevance compared to physical examinations of the animals (Supplementary Materials; Table S1). Although some animals developed abscesses at necropsy (Supplementary Material: Table S2), no blood results suggested generalized septicemia and localized infections. In conclusion, the obtained results indicate that the use of medical glue did not cause any adverse effects in animals that completed the observation period after 3 and 6 weeks.

### Immunohistochemical evaluation of Aggrecan and Collagen II expression

**Figure 6:**
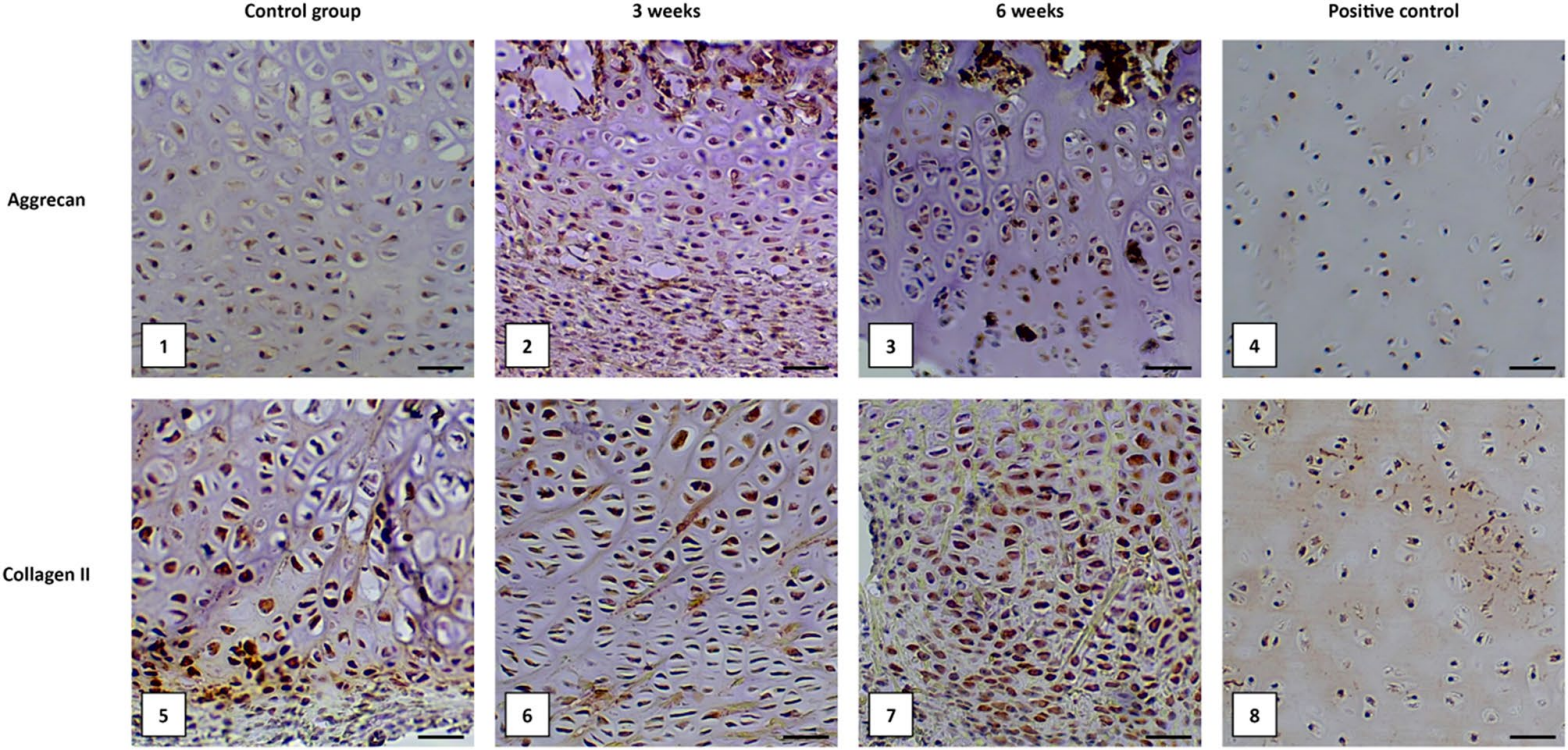
Immunodetection of Aggrecan and Collagen II expression in the extracellular matrix and cytoplasm of chondrocytes in the caudal part of the sternum. Representative samples stained with anti-Aggrecan antibody (**1–4**) and anti-Collagen II antibody (**5–8**) are shown. Similar results were obtained in cranial and medial parts of the sternum. Sections obtained from porcine xyphoid cartilage were the positive control. Scale bars represent 50 μm.

We observed the expression of Aggrecan and Collagen II in the extracellular matrix and cytoplasm of chondrocytes in the growing cartilage in all examined sections of the sternum (Fig. 6).

There were no significant differences between the control and the study group after 3 and 6 weeks from the implantation of medical glue containing allogeneic chondrocytes into the sternum wound after medial sternotomy. We also did not observe differences in cellular and tissue expression of analyzed proteins between different parts (cranial, medial, caudal) of the sternum.

### Implantation of medical glue with allogeneic chondrocytes does not interfere with the healing process of the sternum wound after medial sternotomy

Histopathological examination performed 3 and 6 weeks after the procedure, both in the control group and the study group in terms of the assessment of basic inflammatory parameters and the presence of cellular structures, did not show any significant differences (Table 2).

**Table 2:** List of assessed histopathological parameters.

	Control group	Study group
Quantity	8	10
Inflammatory lesions in soft tissues	Enhanced—4 Insignificant—4	Enhanced—4 Insignificant—6
Resorptive multinucleated histiocytes	1	1
Presence of mature vitreous cartilage (coexistence)	6	8
Polymorphonuclear chondrocytes (coexistance)	3	4
Blurring of layering, polarity of cartilage cell arrangement (coexistance)	1	2
Young cartilage (coexistance)	6	6
Mesenchymal cell concentrations (coexistance)	3	5

**Figure 7:**
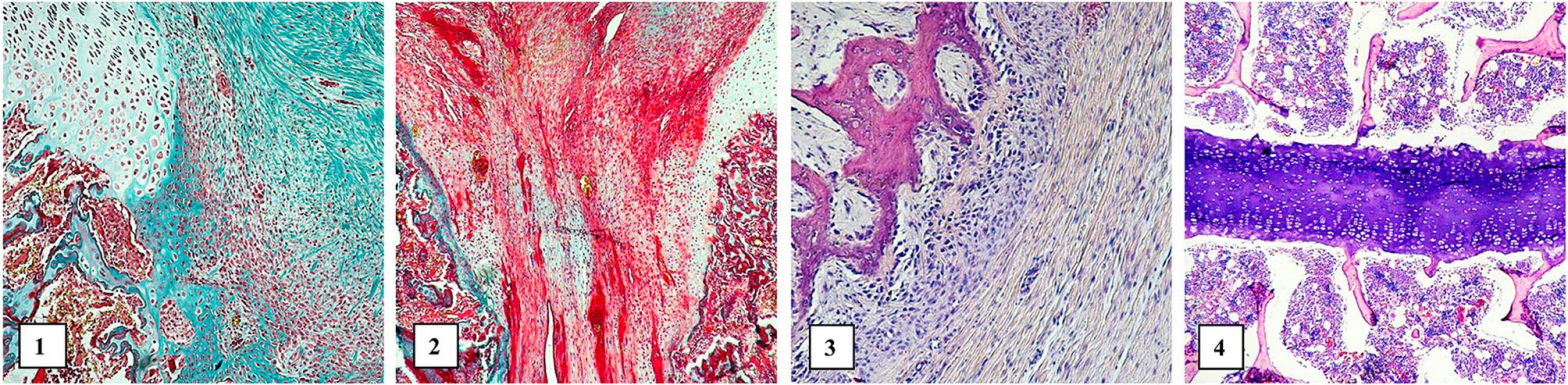
Histopathological analysis in the control group: (**1**)—Masson 100 × Normotypic hyaline cartilage (upper left) in contact with undifferentiated cells, probably of mesenchymal origin, and chondroblasts (lower right). Visible structures of the medullary cavity (lower left). Magn 100-fold. Masson trichrome; (**2)—**Cartilage and fibrous tissues focally mature in the joints of two bone surfaces. Masson trichrome. Magn 100-fold.; (**3**)—thin layer of young chondrocytes lying between the fibrous tissue (right) and bone trabeculae (left). Magn 150-fold. H&E; (**4**)—bar-like structure consisted of mature chondrocytes located in the marrow cavity. H&E. Magn 80-fold. All images were taken under Leica DMLB microscope, USA.

**Figure 8:**
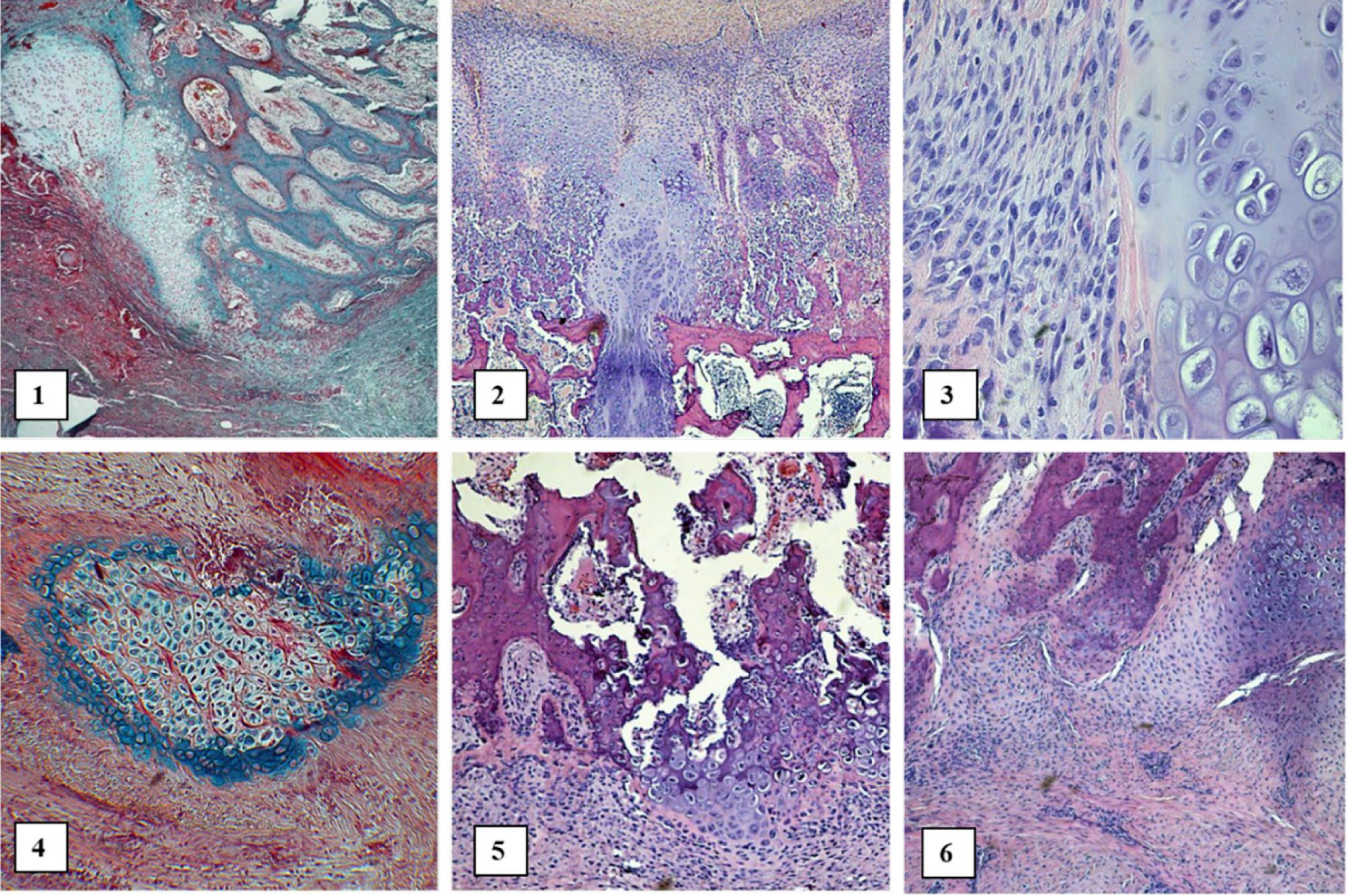
Histopathological analysis in the study group: 3 weeks (1–3), (**1**)**—**Well differentiated cartilage and fibrous tissue overgrows into marrow cavity Magn 50-fold. Masson trichrome; (**2**)**—**well differentiated cartilage penetrates between the two parts of the bone (dissected sterna parts). Magn 50-fold. H&E; (**3**)—well differentiated cartilage and poorly differentiated fibroblast-like cells, probably mesenchymal. Magn 200-fold. H&E; 6 weeks (4–6): (**4**)—chondrocytes nest in soft tissues. Larger amount of bluish mucopolysaccharides are located at the edge of the nest. Magn 100-fold. Movat pentachrome; (**5**)—well differentiated chondrocytes (right) grow into the marrow cavity. On the left and in the middle of the image, poorly differentiated cells, chondroblasts and mesenchymal cells are visible. Magn 100-fold. H&E; (**6)**—normotypic proliferation and ingrowth of chondrocytes into the marrow cavity. Magn 80-fold. H&E.

We observed similar histopathological features of healing in both groups. No cases of cartilage neoplasia were observed, although cases of impaired maturation were seen in both groups—presence of polymorphic chondrocytes and blurred stratification and polarization of chondrocyte arrangement. These lesions coexisted at the periphery of the mature cartilage, so they should be considered as leading to mature cartilage (Figs. 7, 8).

## Discussion

Wound healing of the sternum after median sternotomy, currently the most common method of surgical access to the internal thoracic organs, remains one of the most significant post-operative complications^26,27^, which affects the length of hospital stay and patient mortality^28^. One of the key elements is to achieve rapid synthesis of the sternum that provides adequate stabilization and restores the continuity of its structure, thus reducing the possibility of developing a deep wound infection^6–8,29^.

This seems difficult to achieve due to the impact of the patient’s risk factors, i.e. obesity, age, gender, which impede the proper course of the healing process^30^. It has been shown in literature that the use of existing reconstructive techniques reduces the length of hospital stay, but does not reduce the risk of infection^31^. Furthermore, the use of chondrocyte cells suggests that it may be very beneficial in patients with osteoporosis, in whom the regeneration process is impaired^32^ and in patients with a paramedial sternotomy wound requiring rapid and effective stabilization^33^. A promising solution could be the use of cell cultures, in particular chondrocytes. Under the right conditions, these cultures are successfully used in the treatment of cartilage defects in knee joints and osteoarticular lesions^34,35^.

The extraction of chondrocyte cells from articular cartilage, auricle or trachea for the regeneration of cartilage defects is an increasingly used technique^36–38^. It has been shown in the literature that it is possible to obtain viable chondrocytes using a digestion medium based on collagenase II at concentrations in the range of 0.1–0.2%^38^, which was confirmed in our study (Fig. 1). In addition, significant differences in the level of cell proliferation were observed depending on the culture medium used^39^. This level was highest for the commercial and self-prepared pig serum compared to the commonly used FCS and FBS serum (Fig. 4). Clear differences in cell phenotype were also observed. This shows what effect the use of specific cell medium has on the yield of the obtained culture.

Chondrocyte cultures are characterized by the expression of characteristic genes: Collagen II, Aggrecan and Sox9, which allows for their direct detection in cell cultures using immunocytochemical techniques^40–43^. Our analysis confirmed their presence. It was also shown that the levels of the identified markers varied depending on the passages (Fig. 2). This correlation of the decrease in the number of identified protein products with successive passages in the chondrocyte culture was observed by RT-PCR gene expression analysis and the presence of gene protein products. There was a high level of gene expression after the first passage (P1), which decreased in the two subsequent passages assessed—P2 and P3 (Figs. 3, 5), as confirmed in the literature^44,45^. This demonstrates susceptibility to dedifferentiation in later passages and reduced proliferative capacity of chondrocyte cultures.

Of key importance in our study was the fact that the obtained cell cultures showed the ability to proliferate after transplantation into the sternotomy wound. It has been previously shown that chondrocyte cells can be transplanted into knee joints and heal the injured area^46^. The cells actively proliferated and formed an extracellular matrix^43^. This effect was also observed in the sternal tissue we studied. Immunohistochemical and histopathological analysis showed the presence of chondrocytes in the formed wound (Figs. 6, 7, 8). They show typical morphological features of chondrocytes and the presence of characteristic protein products, i.e. Collagen II and Aggrecan. These products were observed both 3 weeks and 6 weeks after implantation.

To the best of our knowledge, the application of allogeneic chondrocytes to a sternal wound has been used for the first time in the treatment of a median sternotomy wound. This method appears to have great potential, as it rapidly fills the surgical wound, thus closing the space for the penetration of infection-causing microorganisms. Infections of deep sternal wounds and their long healing time remain a serious problem, contributing to prolonged hospitalization of patients, increasing treatment costs, and worse, being one of the life-threatening factors.

It is possible to obtain a viable culture of porcine chondrocytes from the myeloid process, which is influenced by the type of serum supplemented. The isolated cells show phenotypic stability up to 3 passages after isolation. Application of the cells in tissue glue directly to the sternotomy wound does not cause inflammation or necrosis, which allows us to conclude that this method is safe. We believe that the use of chondrocytes in tissue glue as an innovative method of treating sternotomy wounds will accelerate research and bring us closer to reducing infection of sternotomy wounds.

Nevertheless, our study has several limitations. Firstly, the animals used were completely healthy with no risk factors such as obesity, diabetes or age, that could affect the rate of wound healing. In addition, the animal model used moves on four limbs (front and back limbs), which causes a greater load on the sternum after surgery, which has no correlation with the post-operative situation in humans. Moreover, the metabolism and biological processes in animals are much faster than in humans, so it is impossible to accurately calculate whether the observation period we chose was valid. This requires further evaluation using older animals and additional time points.

Further studies evaluating the presence of chondrocytes implanted into the sternotomy wound at longer time points are needed to assess cell maturity and quantify the continued effectiveness of the method.

## Methods

### Animal studies

**Figure 9:**
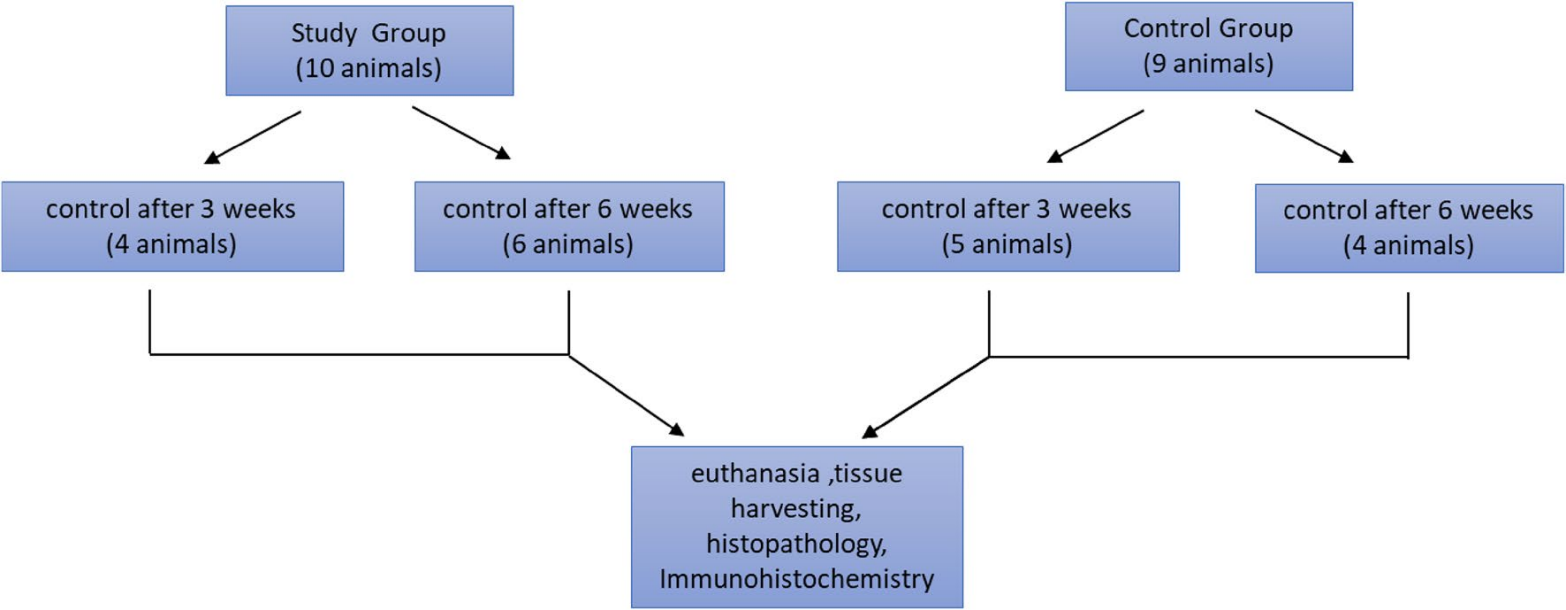
Study workflow.

This part of the project was carried out at the Center for Cardiovascular Research and Development of American Heart of Poland. All methods were performed in accordance with the Animal Welfare Act and the “Guide for the Care and Use of Laboratory Animals”. All procedures were approved by the Local Ethics Committee at the Medical University of Silesia in Katowice (Decision No. 61/2018). All methods are reported in accordance with ARRIVE guidelines (https://arriveguidelines.org). A total of 19 domestic swine (*Sus scrofa domesticus*), with an average body weight of 40 kg, were incorporated in this study. Animals were divided into the control and study groups for 3 and 6 weeks of observation (Fig. 9).

### Anesthesia, monitoring and emergency procedures

In order to maintain general anesthesia, Isoflurane (Aerrane, Baxter, Poland) was used at a concentration of 1–3% in a mixture with oxygen (50–100%) or alternatively, a continuous drip infusion of Propofol (Braun, Poland) was administered in an amount of approx. 12–20 mg/kg/h. During the procedure, basic physiological parameters such as ECG, arterial pressure, respiratory frequency and saturation were constantly monitored. Additionally, at the discretion of the anesthetist, the following were also administered: Fentanyl (Fentanyl WZF—50 µg/mL, WZF, Poland) at a dose of 2–4 µg/kg b.w IV by bolus or infusion at the rate of 0.03–0.06 µg/kg/min.

### Median sternotomy

After achieving a proper level of anesthesia, the surgical area was roughly cleaned and disinfected. Midline incision of the skin and subcutaneous tissues was performed and the sternum was exposed. With the help of an electric saw, the sternum was incised over the midline. The wound remained open for 60 min. In the control group, the sternum was closed with surgical wires, and the skin and subcutaneous tissues were closed with sutures. The study group received the medical glue with allogeneic chondrocytes on the borders of the sternum’s wounds, the sternum was closed in the same manner as the control group, and another layer of medical glue with allogeneic chondrocytes was applied over the junction in the wound. The skin and subcutaneous tissues were closed in the same manner as in the control group. A second dose of antibiotics (ceftiofur) was administered (Cemay, Livisto, Poland) 3–4 mg/kg IM. Once the procedure was finished, the surgical wound was sutured.

### Blood collection, euthanasia and tissue harvesting

Blood ^20ml^ was collected before performing sternotomy and euthanasia procedures on EDTA (10 ml) and a container without additives (10 ml) (for plasma and serum, respectively), centrifuged 5 min/3000–4000 RPM, transferred to cryotubes and frozen at -80 °C. Animals were euthanized with a commercially available solution of sodium pentobarbital (Morbital, Biowet Puławy, Poland) 1–3 ml/10 kg b.w. IV under general anesthesia. Death was confirmed by pupil dilation, cardiac and respiratory cessation, and lack of reflexes to stimuli. Immediately, the treated area was aseptically prepared before collection of the samples. The sternum was exposed and a total of 6 samples (2 × cranial, 2 × medial, 2 × caudal) were taken. One sample from each region was fixed in 10% NBF (Neutral Buffered Formalin) and the other sample was fixed in 70% alcohol.

### Tissue processing

Samples fixed in 10% NBF were cut into microscopic sections. Necrosis, inflammation, tumor foci, fibrin were assessed in the obtained preparations. This allowed the evaluation of the reaction of the surrounding tissues and the healing of the implanted cells. Samples fixed in 70% alcohol were immediately transported to the immunohistochemistry laboratory within 48 h. The presence of chondrocytes in the biopsy was confirmed by immunohistochemistry using labeled antibodies. An additional test confirming the presence of implanted chondrocytes in the examined tissue was the evaluation of the expression of Collagen II and Aggrecan, which are protein products of genes.

### Immunocytochemistry

Cells were seeded in 24-well plates at a density 4 × 10^5^ cells per well. After overnight incubation at 37 ºC and 5% CO_2_, cells were stained with 4% paraformaldehyde (Sigma Aldrich, Darmstadt, Germany) for 10 min. Subsequently, cells were washed 3 times with DPBS and blocked with solution of 1% milk in PBST (PBS with 0.05% Tween20) for 30 min at room temperature (RT). Cells were stained with primary antibody (1:100) overnight in 4 ºC following washing with PBST solution. After the incubation time, the cells were stained with secondary antibody (1:500) for 1 h at RT. The nuclei were stained with 300 nM of DAPI solution (2-[4-(Aminoiminomethyl)phenyl]-1H-Indole-6-carboximidamide hydrochloride) in the dark (Sigma Aldrich, Darmstadt, Germany). Cells were stained with Collagen II, Aggrecan and Sox9 following washing 3 times with DPBS. Cells were visualized under fluorescence microscope (OLYMPUS, Japan). Antibodies used in immunofluorescence are listed in Table 3.

**Table 3:** Antibodies used for immunofluorescence staining.

Antibody	Dilution	Company Cat #
Anti-Collagen II antibody	1:100	Abcam Cat#ab34712
anti-Aggrecan (ACAN) (N-Term) antibody	1:100	ABIN2707311
Anti-SOX9 antibody	1:100	AB185966-100
Goat anti-Mouse IgG (H+L) Highly Cross-Adsorbed Secondary Antibody, Alexa Fluor™ 488	1:500	ThermoScientific Cat#A11029
DAPI	300 nM	VECTASHIELD H-1200

### Chondrocytes isolation

The porcine xiphoid cartilage was harvested under aseptic condition. The tissue was placed in cell culture medium consisting of DMEM/F12 (PAN Biotech, Germany), 10,000 U/ml Penicillin, 10 mg/ml Streptomycin, 25 μg/ml Amphotericin B (PAN Biotech, Germany) and transported to the laboratory. Subsequently the tissue was washed by Dulbecco’s Phosphate Buffered Saline w/o calcium, magnesium (DPBS) (PAN Biotech, Germany) and minced into 1 mm^3^ pieces. The next step was to digest the tissue by 0,6% collagenase II (Sigma aldrich, Darmstadt, Germany) overnight at 37 ºC and 5% CO_2._ The following day the sample was filtered through cell strainer 70 μM (ThermoScientific, MA, USA). Cells suspension was centrifuged at 500×g for 5 min. Afterwards, supernatant was aspirated and the pellet was resuspended in DPBS. Isolation efficiency was calculated by determining the cells density and viability using Automated Cell Counter TC20 (Bio-Rad, USA).

### Cell culture

To optimize chondrocytes cell culture conditions, cells were seeded in four cell culture media. Each media contained the same among of antibiotics (Penicillin 10,000 U/ml, Streptomycin 10 mg/ml, Amphotericin B 25 μg/ml), Vitamin C, DMEM/F12. Each culture media were prepared using different types of serum:10% FBS, 10% commercial pig serum (pig com), 10% self-prepared pig serum (self-pig) and FCS. Chondrocytes were seeded at a density 1 × 10^6^ cells/T-75 flask in four different culture media. Cells were cultured until third passage, samples were taken for analysis after each passage.

### Cells proliferation assay

Cells were seeded at a density 1.5 × 10^4^ cells per cm^3^ and incubated 24 h in 37 ºC, 5%CO_2_. The cell proliferation assay was performed according to the manufacturer’s instructions (Promega, Wisconsin, United States). The measurement was performed on a day 0 (control), 1, 2 and 3 in 96-well TPP™ plate (PerkinElmer, Waltham, MA, USA). The intensity of the fluorescence emission was detected at 590 nm using a VICTOR™ Multilabel Plate Reader (PerkinElmer, Waltham, MA, USA) with a 560 nm excitation source.

### Western blotting

Cells pellets were dissolved in lysis buffer (1 mM EDTA, 0,1% TritonX-100 with addition of Halt Protease Inhibitor Coctail (ThermoScientific, MA, USA), incubated for 30 min on ice (with vortexing every 5 min). Protein concentration was measured using the Commasie (Bradford) Protein Assay (Thermofisher, MA, USA) according to supplier’s protocol. 5 × Laemmli Buffer with 5% β-mercaptoethanol was added to the samples and denatured for 10 min at 95 °C. Samples (7 µg of proteins) were subjected to standard SDS PAGE electrophoresis on 5% stacking and 10% separating gel and then transferred to a PVDF Immobilon membrane (GE Healthcare) using the “mini PROTEAN system” (Bio-Rad, USA).

After protein transfer, the membranes were cut to 2 or 3 pieces based on the molecular weight to allow simultaneous use of different antibodies corresponding to proteins with different molecular weights. The size of protein products was estimated using the PageRuler™ Prestained Protein Ladder size marker, 10–180 kDa (Thermo Scientific™, 26616). Then, membranes were blocked in 1,5% dry milk solution in TBST buffer (0,05% Tween20) for 2 h at RT. Subsequently, membranes were incubated overnight at 4 °C with primary antibody anti SOX 9 (1:500) (Biorbyt, orb4387), anti GAPDH (1:250) (Biorbyt, orb88269), Anti Collagen II (1:1000) (Biorbyt, orb10436) and Anti Aggrecan (1:1000) (Abbiotec, 251591). The membranes were then incubated with secondary anti-rabbit IgG antibodies (IgG Whole Molecule) conjugated with horseradish peroxidase (Horseradish Peroxidase Conjugated) at a dilution of 1:15,000. Secondary antibodies were detected by incubating the membrane in Pierce 1-step Ultra TMB Blotting Solution (ThermoScientific, MA, USA) for 30 min at RT.

### RNA Isolation and Quantitative RT-PCR

Total RNA was isolated using the RNeasy Plus Mini Kit (Qiagen, Germany) according to the manufacturer’s protocol. cDNA was synthesized from 2 µg RNA with the Revert Aid First Strand cDNA Synthesis Kit (Thermo Scientific, MA, USA) according to the manufacturer’s instructions. Relative expression levels were measured in triplicates in a Roche Light Cycler 480 using Power SYBR Green PCR Master Mix (Applied Biosystems, Germany), 300 mM primers (Supplementary Materials; Table S3) and 1/15 cDNA stock. Values were calculated using the Pfaffl method and normalized to those of GAPDH.

### Statistical analysis

Statistical analyses of the qRT-PCR data were performed with Microsoft Excel software. Normalized relative expression levels were used to calculate the mean and the SEM of all experiments, represented by columns and error bars on the figures, n = 3.

### Histopathological examination

MOVAT-Russel Pentachrome, hematoxylin & eosin (H&E) and Masson–Goldner Trichome staining (DiaPath, Italy) were performed according to the manufacturer’s instructions.

### Immunohistochemistry

Paraffin blocks containing pieces of the pig's sternum were cut on a semi-automatic microtome HM 340E (ThermoScientific) into serial sections with a thickness of 4 µm. The obtained sections, after dewaxing and hydration were subjected to the antigen (aggrecan and collagen II) immunodetection. The antigen retrieval process was carried out with a citrate buffer, pH 6 (Vector Laboratories) for 30 min in a water bath, at 95 °C. Endogenous peroxidase activity was blocked with 3% H_2_O_2_ for 10 min and non-specific binding sites were blocked by incubation with 10% horse serum (Vector Laboratories) for 60 min. To detect aggrecan, sections were incubated with the anti-Aggrecan antibody (NB600-504; Novusbio) at a dilution of 1:200 (2 h at room temperature, followed by 18 h at 4 °C). After washing the slides, secondary antibody conjugated with horse radish peroxidase (ImmPRESS Horse Anti-Mouse IgG Polymer Kit Peroxidase; Vector Laboratories) was applied. The negative control was monoclonal Mouse IgG (Isotype Control ab18443; Abcam), diluted to the same concentration as the primary antibody. Collagen II were identified in the sternum with anti-Collagen II antibody (ab 34712; Abcam) which was applied at a dilution of 1:200, for 20 h at 4 °C. Then, the secondary horse radish peroxidase-conjugated Rabbit antibody (ImmPRESS Horse Anti-Rabbit IgG Polymer Kit Peroxidase; VectorLaboratories) was applied. The negative control was a monoclonal Rabbit IgG (Isotype Control 3900; Cell Signaling Technology), diluted to the same concentration as the primary antibody. Positive IHC reactions were visualized by using diaminobenzidine (ImmPACT DAB; Vector Laboratories), a substrate for peroxidase, applied to the sections for 1 min. The brown product formed in this reactions indicated the location of aggrecan or collagen II, respectively. Sections were then counterstained with Gill’s hematoxylin (Vector Laboratories). Positive control sections was porcine xyphoid cartilage taken from healthy pig. The obtained IHC stains were subjected to qualitative assessment. Photographic documentation was made for each section at magnification 100×. The location of aggrecan and collagen II expression in the examined tissues was assessed.

### Interleukins and TNF concentration measurements

The concentration of interleukin 1 alpha (IL-1 alpha), interleukin 6 (IL-6) and Tumor Necrosis Factor (TNF) were measured using the Porcine solid-phase sandwich ELISA (enzyme-linked immunosorbent assay) (ThermoScientific, MA, Germany). It was designed to measure the amount of the target bound between a matched pair of antibodies. A target-specific antibody has been pre-coated in the wells of the supplied microplate. Samples, standards, or controls were then added to these wells and bound to the immobilized (capture) antibody. The sandwich was formed by adding the second (detector) antibody, a substrate solution was added that reacts with the enzyme-antibody-target complex to produce measurable signal. The intensity of this signal was directly proportional to the concentration of the target present in the original sample.

### Ethical approval

The animal study protocol was approved by Local Ethics Committee for animal research (Contract No. 61/2018).

### Supplementary Information

Supplementary Figure S1.

Supplementary Tables.

## Data Availability

The datasets used and/or analyzed during the current study are available from the corresponding author on reasonable request.
