# Supplementary material for: Impact of local delivery of allogeneic chondrocytes on the biological response and healing of the sternum bones after sternotomy
Source: Sci Rep. 2023 Sep 25;13:15971. doi: 10.1038/s41598-023-43255-y (PMC10520054; doi:10.1038/s41598-023-43255-y)

**Supplementary Figure S1.** Original blots. Membrane fragments immunoblotted respectively: A: Collagen II; B: GAPDH; C: SOX 9; D: Aggrecan. Identification of Sox9, Collagen II and Aggrecan in swine chondrocytes cultured in different serum at passage 0, 1, 2 and 3. The size of protein products was estimated using the PageRuler™ Prestained Protein Ladder size marker, 10 to 180kDa (Thermo Scientific™, 26616).


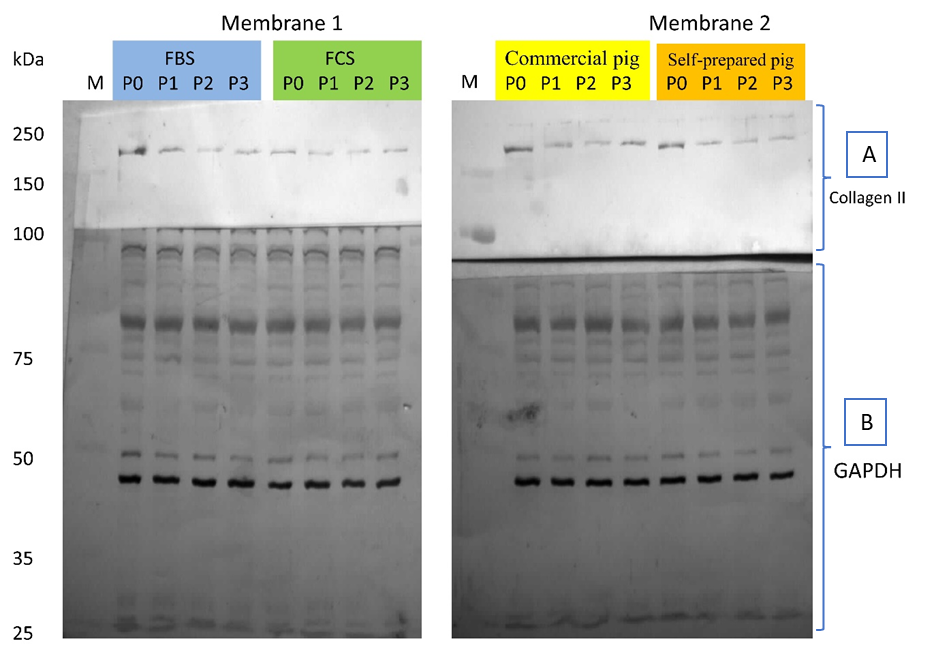


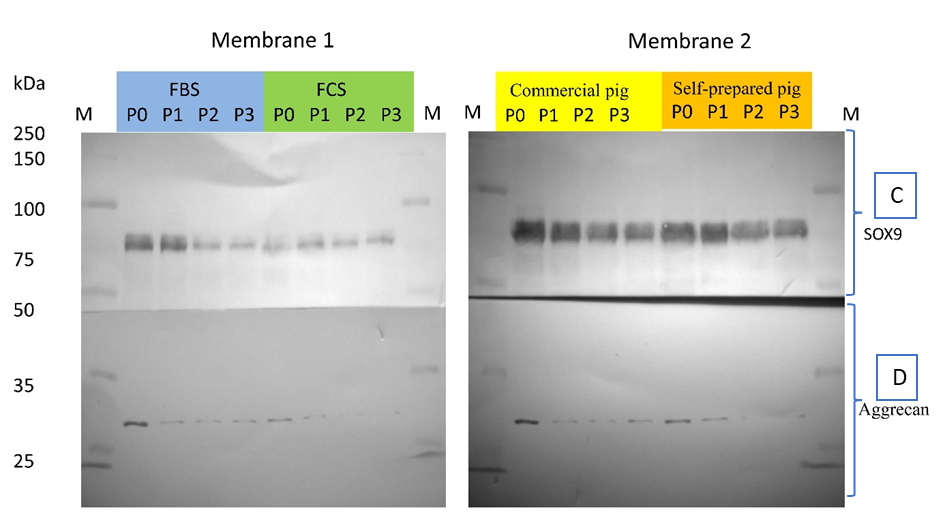

Supplement: Supplementary file 1 — Supplementary Figure S1. [file 41598_2023_43255_MOESM1_ESM.docx]
