# Supplementary material for: Impact of local delivery of allogeneic chondrocytes on the biological response and healing of the sternum bones after sternotomy
Source: Sci Rep. 2023 Sep 25;13:15971. doi: 10.1038/s41598-023-43255-y (PMC10520054; doi:10.1038/s41598-023-43255-y)
Supplement: Supplementary file 2 — Supplementary Tables. [file 41598_2023_43255_MOESM2_ESM.docx]

**Supplementary Table S1.** Results of complete blood count (CBC) and biochemical analysis in the study and control groups after 3 and 6 weeks of observation.

| 3 Weeks | **COMPLETE BLOOD COUNT (CBC)** | **Mean** | **SD** | **p value** |  |
| --- | --- | --- | --- | --- | --- |
|  |  |  |  |  |  |
|  | White Blood Cells (WBC) | 19,6 | 5 | 0,8 |  |
|  | Red Blood Cells (RBC) | 6,6 | 0,5 | 0,27 |  |
|  | Hemoglobin (Hg) | 10,3 | 0,6 | 0,44 |  |
|  | Hematocrit (Htc) | 33,9 | 1,9 | 0,9 |  |
|  | Mean Corpuscular Volumen (MCV) | 51,6 | 3,5 | 0,15 |  |
|  | Mean Corpuscular Hemoglobin (MCH) | 15,6 | 1,2 | 0,05 |  |
|  | Mean Corpuscular Hemoglobin Concenration (MCHC) | 30,4 | 0,6 | 0,01 |  |
|  | Platelets (PLT) | 484,6 | 145,7 | 0,04 |  |
|  |  |  |  |  |  |
|  | **BIOCHEMISTRY** |  |  |  |  |
|  | Alanine Aminotransferase (ALT) | 38,4 | 14,2 | 0,03 |  |
|  | Aspartate Aminotransferase (AST) | 32,2 | 8,9 | 0,03 |  |
|  | Creatinine | 115 | 13,7 | 0,29 |  |
|  | Urea | 3,3 | 0,7 | 0,13 |  |
|  | Alkaline Phosphatase (ALP) | 126,4 | 37,7 | 0,75 |  |
|  | Albumin | 33,5 | 3,3 | 0 |  |
|  | Total Protein (TP) | 72 | 4,2 | 0,76 |  |
|  | Glucose | 5,3 | 0,8 | 0,4 |  |
|  | C-Reactive Protein (CRP) | 0,3 | 0,1 | 0,01 |  |
| 6 Weeks | **COMPLETE BLOOD COUNT (CBC)** | **Mean** | **SD** | **p value** |  |
|  |  |  |  |  |  |
|  | White Blood Cells (WBC) | 17,7 | 6,06 | 0,55 |  |
|  | Red Blood Cells (RBC) | 6,6 | 1,66 | 0,78 |  |
|  | Hemoglobin (Hg) | 10,1 | 2,48 | 0,73 |  |
|  | Hematocrit (Htc) | 33,2 | 8,54 | 0,75 |  |
|  | Mean Corpuscular Volumen (MCV) | 49,8 | 2,36 | 0,96 |  |
|  | Mean Corpuscular Hemoglobin (MCH) | 15,3 | 0,94 | 0,76 |  |
|  | Mean Corpuscular Hemoglobin Concenration (MCHC) | 30,8 | 1,47 | 0,77 |  |
|  | Patelets (PLT) | 316,2 | 152 | 0,61 |  |
|  |  |  |  |  |  |
|  | **BIOCHEMISTRY** |  |  |  |  |
|  | Alanine Aminotransferase (ALT) | 60,6 | 16,4 | 0,94 |  |
|  | Aspartate Aminotransferase (AST) | 111,2 | 221 | 0,42 |  |
|  | Creatinine | 119,7 | 18,9 | 0,16 |  |
|  | Urea | 4,5 | 0,99 | 0,62 |  |
|  | Alkaline Phosphatase (ALP) | 223 | 143 | 0,32 |  |
|  | Total Protein (TP) | 75,1 | 8,16 | 0,05 |  |
|  | Albumin | 35,6 | 4,26 | 0,4 |  |
|  | Glucose | 4,8 | 0,68 | 0,09 |  |
|  | C-Reactive Protein (CRP) | 0,3 | 0,15 | 0,41 |  |

**Supplementary Table S2.** The presence of encapsulated superficial abscesses.

| **Study Group** | **Presence of superficial abscesses** | |
| --- | --- | --- |
|  | **3 weeks** | **6 weeks** |
| **Control** | 2 | 0 |
| **Treatment** | 0 | 2 |

**Supplementary Table S3. Primers used in qRT-PCR analysis.**

| **Gene** | **Forward primer (5’-3’)** | **Reverse primer (5’-3’)** |
| --- | --- | --- |
| **Collagen II** | 5’GGAGCAGCAAGAGCAAGGA3’ | 5’GCAGTGTTAGGAGCCAGGTT3’ |
| **Aggrecan** | 5’CGTGGTCCAGCACTTCTAAA 3’ | 5’AGTCCACTGAGATCCTCTAC3’ |
| **Sox9** | 5’TGCTGAATGAGAGCGAGAAG3’ | 5’CGCGGCTGGTACTTGTAAT3’ |
| **GAPDH** | 5’ACCTCCACTACATGGTCTACA3’ | 5’ATGACAAGCTTCCCGTTCTC3’ |
